# A description of events surrounding the index COVID-19 diagnosis in a staff member at Kalafong Provincial Tertiary Hospital in Gauteng, South Africa

**DOI:** 10.4102/safp.v62i1.5196

**Published:** 2020-11-16

**Authors:** Indiran Govender, Keorapetse A.O. Masilo, Olga M. Maphasha, Sello Matjila

**Affiliations:** 1 Department of Family Medicine, University of Pretoria and Kalafong Provincial Tertiary Hospital, Pretoria, South Africa

**Keywords:** Kalafong Hospital, Tertiary Hospital, Gauteng, SARS-CoV 2, COVID-19

## Abstract

The ongoing coronavirus disease 2019 (COVID-19) pandemic presented a huge challenge to the health systems across the world. When the virus hit South Africa, and the state of national disaster was announced by the president, the healthcare system had to work on its COVID-19 response preparedness. Initially, a few hospitals were then designated facilities for managing COVID-19 patients. Kalafong Hospital, which was not amongst a list of designated facilities for COVID-19 was forced to evaluate its level of preparedness after an intern doctor tested positive. The objectives of this report are to illustrate the hospital’s response around the management of the index case to share our facility’s general response to the pandemic.

## Introduction

On 11th March 2020, the World Health Organisation (WHO) declared the coronavirus disease 2019 (COVID-19) outbreak a global pandemic.^1^ At the time of the announcement, South Africa had recorded a total number of 13 cases.^2^ The first reported case at Kalafong Provincial Tertiary Hospital (KPTH) was a member of staff who tested positive for COVID-19 on 14th March, 7 days after the first case in Gauteng Province was reported.^3^

Kalafong Provincial Tertiary Hospital is located in the southern part of Tshwane district. The 859 bedded hospital serves an estimated population of two million people.^4,5^ One of the major concerns for the National Department of Health (NDOH) was the healthcare system being overwhelmed and exhausted by cases requiring hospitalisation and intensive care. Long-term projections from NDOH’s primary model estimated that 20 000–30 000 patients might require intensive care. This is an alarming number as the entire country’s Intensive Care Unit (ICU) bed capacity is estimated to be 3300.^6^

As early as February, the hospital set up a COVID-19 task team to prepare for the pandemic. The task team comprised of representatives from nursing, medical, pharmacy, infection control, occupational health, quality control, clinical managers, supply chain, cleaning, security and labour. The following measures were implemented to allay staff anxiety, to prevent primary infection in patients and staff members and to empower and facilitate confidence amongst members of staff. These include restriction of visitors, limiting the hospital for patients and visitors entrance to a single point, preparing isolation units in the emergency department, designating wards to manage COVID-19 patients, ordering personal protective equipment (PPE), implementing screening of patients, security staff spraying hand sanitisers on all staff members entering the hospital premises, hand sanitisers being placed at the entrance of each ward, and multiple education and training workshops were done for all members of staff. The biometric security systems leading to the University satellite campus were also disabled.

## Perspective

An intern doctor working in the surgery department visited friends at the airport who were arriving from Europe. She subsequently developed flu-like symptoms and with advice from her General Practitioner tested for COVID-19. Her results turned positive for COVID-19 and the relevant authorities were notified.

A total of 56 staff members identified themselves as contacts. They all self-quarantined at home for 14 days. Other healthcare workers who were not included in the identified 56, including those without symptoms and no contacts then insisted to be tested for COVID-19. Informal conversations during meetings with those affected revealed they were anxious and afraid.

As an index case, challenges including stigmatisation arose as many staff members were still unfamiliar with the condition. The doctor reported the stigma of having COVID-19 as being worse than the virus itself.^7^

The hospital was in the process of emptying the two surgical wards as a part of the plan to decongest the hospital and to create wards to admit COVID-19 patients. This fed into the anxiety as staff felt that the two surgical wards were subsequently closed as a response to the COVID-19 infection. All healthcare workers including majority of doctors and nurses who had worked in those wards between Thursday and Friday were sent home for self-quarantine. This resulted in nurses from other wards being redeployed to the two wards so that patient care and service delivery could continue. Further hospital service interruption included cancellation of elective surgeries and follow-up dates being given to patients. Hospital management worked extremely hard to allay the anxiety of staff. It was an opportunity to learn more about the disease.

This incident exposed gaps in the hospital preparedness and helped the hospital to rectify these plans. Since this incident, two screening tents have been placed at the entrance of the hospital. All patients upon arrival go through primary screening using a screening tool that identifies the common symptoms experienced in COVID-19 as per NICD guidelines. Once a patient has a positive screening test, they get secondary screening done in another tent by doctors. All patients fulfilling criteria for person under investigation proceed to the testing site where they are tested for COVID-19. The system has been efficient because of consistency and dedication by the front-line staff. Since the beginning of the pandemic, the number of screenings done per day has increased. The hospital screens an average of 950 patients per day. All staff members are screened daily before the beginning of each shift. Over 1000 staff members have been recorded as being screened daily. Those with symptoms are required to notify their supervisors prior to the shift and arrangements are then made for them to get tested. After testing they immediately go home for self-isolation. Thirdly, a different entry port into the emergency department has been created. This ensures that suspected cases are isolated from the rest of the main casualty. All patients and members of staff entering the hospital premises are required to wear face masks and flu vaccines were offered to all healthcare workers.

Departmental morning meeting times were reduced with some departments cancelling academic presentations to decrease the time spent in the meeting. Training workshops were increased and made mandatory for members of staff to attend. The workshops served as an educational platform and were used to empower staff members with proper hand washing techniques, infection control measures and the right way to use PPE. These sessions are aimed at improving knowledge, skills and attitudes towards COVID-19 disease. The ultimate objective is to reduce anxiety and empower the hospital healthcare workers. Workers that have been properly prepared for the job and the challenges associated have a reduced risk of mental-health problems.^8^ Communication was enhanced by moving to virtual platforms for meetings, bulk sms messages, creation of WhatsApp groups and creation of a newsletter.

## Discussion

Whilst hospitals were preparing for COVID-19 pandemic, no one could have predicted the number of outbreaks that occurred within hospitals in the initial stages of the pandemic. One of the first hospitals in South Africa to be affected by the COVID-19 pandemic was a private hospital situated in Durban.

A study was conducted to investigate the extent of the outbreak and the most probable cause of events that resulted in the spread of the virus within a private facility, in KZN. From the results, there were 119 confirmed cases which included 39 patients and 80 members of staff. Fifteen of the 39 patients died resulting in a case fatality rate of 38.5%. This resulted in closing the hospital in an attempt to stop the virus from spreading to the rest of the staff and patients. This outbreak also led to clusters of COVID-19 cases in a local nursing home and an outpatient dialysis unit of the hospital campus resulting in 17 cases.^9^ Tygerberg hospital in the Western Cape and Morningside Mediclinic in Gauteng were amongst those hospitals that had increased number of staff members with infections in Tygerberg to date, recording a total of four staff member deaths.^10^

Timeous management of a COVID-19 suspect or case is important in preventing an outbreak. The hospital’s management responded to the case swiftly and the following measures were taken. The doctor involved was advised to self-isolate at home for 14 days and was requested to use a 7-day symptom screening tool. This is in accordance with the NICD guidelines for quarantine and isolation in relation to COVID-19 exposure and infection.^11^ The management together with the COVID-19 task team immediately set a meeting to discuss the way forward, establish the doctors’ contacts including family, friends, colleagues, nurses and patients the doctor had managed. Nursing staff and medical doctors from the two wards where the doctor worked on Thursday and Friday were notified by the nursing and clinical manager. Those who were on duty for those days identified themselves as contacts. The challenge experienced was identifying true contacts as defined by NICD guidelines. Some were uncertain if they had come into contact with the doctor whilst others verbalised, they might have shared the same space with her at some point. As a result, it was decided that all members of staff that worked in those wards during the 2-day period should go on self-quarantine for a period of 14 days. Those who had symptoms and those who later developed symptoms all tested negative for COVID-19. All patients admitted to the two affected wards were notified and kept for an extended period of 14 days from the time the doctor’s diagnosis was known. Daily symptom screening on those patients was performed. Patients discharged were contacted by district officials and advised to self-quarantine at home for 14 days and should come back for testing if they develop symptoms.

Infectious disease outbreaks such as COVID-19 can cause feelings of distress and anxiety even in people who are not at high risk of getting sick.^12^ A survey was conducted in China during the beginning stages of the outbreak. The results showed that 53.8% of participants rated the psychological impact of the outbreak as moderate to severe, 16.5% reported moderate to severe depressive symptoms, 28.8% reported moderate to severe anxiety symptoms and 8.1% reported moderate to severe stress symptoms.^13^ Anxiety was the leading symptom experienced by the general population. A different study including 470 healthcare workers analysed the psychological impact the pandemic had on healthcare workers. Medical health workers were compared to non-medical healthcare workers. Fourteen and half per cent of participants screened positive for anxiety, 8.9% for depression and 7.7% screened for clinical post-traumatic stress disorder (PTSD). The prevalence of anxiety was high in both groups but higher in the non-medical group.^13^ These findings are consistent with studies conducted in many other countries which associate pandemics with heightened levels of anxiety and stress.^14^ Healthcare workers are vulnerable to emotional distress given their risk of exposure to the virus, shortages of PPE, concerns about infecting and caring for their loved ones, longer working hours and involvement in emotionally and ethically challenging resource-allocation decisions.^15^

A systemic review on the impact of the pandemic on the mental health of healthcare workers identified lack of support, communication, maladaptive coping and lack of training as common risk factors for developing psychological morbidities.^16^ Protocols and precautionary measures instituted should be clear and support from colleagues and supervisors should always be made available to all staff members.^17^ Adequate training of all staff members is important and a critical factor in ensuring healthcare workers respond to the crisis appropriately. During an outbreak, health facilities are likely to focus on the medical and infection prevention aspects of the disease; however, equal attention should be given to the psychological impact that can be experienced by the patients and staff members. Support groups or services should be made available where a staff member may be able to get assistance without being judged or stigmatised. It has been reported that some people who have been infected or quarantined may experience stigma, guilt or shame.^18^ The stigma could be from the work environment or at community level. Whilst it might be difficult to create a work-friendly environment in the midst of a pandemic, which may result in health systems being fully stretched and others collapsing, healthcare workers’ mental health and morale should be safeguarded as this can either positively or negatively influence the quality of service delivery.^19^ History has shown that most pandemics last more than a year, therefore continual training, education, hygiene reinforcements and support are critical to the continual delivery of quality health services whilst maintaining stringent infection prevention and control measures.

When members of staff test positive for COVID-19 disease, service delivery can be impacted. As seen at Kalafong Hospital, resultant shortages in the workforce can become significant. Because of an unpredictable level of disruption that may occur when a member(s) test positive, staff flexibility, cooperation and clear communication from the hospital directives are essential in ensuring that the hospital continues to function at its highest capacity as possible whilst managing the ongoing cases within the hospital.

The global impact of COVID-19 has led to some institutions implementing extreme measures to ensure that staff shortages are minimal. This includes temporary halting of junior doctors’ rotations, annual leave for staff members being delayed and redeployment of doctors undertaking research activities.^20^ Whilst efforts are made to decrease staff shortages, longer working hours in a stressful environment may potentiate increased levels of stress. This may inversely result in workers that are fatigued, stressed and anxious. Creating platforms to address potential sources of anxiety should be considered as one of the important measures in the fight against COVID-19. Testing of asymptomatic workers could also alleviate a critical source of anxiety.^21^ The health of healthcare workers should be prioritised to ensure uninterrupted quality service delivery. A COVID-19 free workforce that is not burned out will be an asset to the prolonged response to the COVID-19 crisis.^20^

The balance between professional duty, selflessness and personal fear for oneself and others can often cause conflict and dissonance in many healthcare workers.^22^ Regardless of risk of exposure, people may experience fear and anxiety of falling sick or dying, helplessness or blame of other people who are ill, potentially triggering a mental breakdown.^23^

## Conclusion

The hospital response to the COVID-19 response has demonstrated that teamwork, clear guidelines and protocols are key to ensure smooth running, even during a crisis. Consideration should be made for the heterogeneity of the hospital staff during engagements, and when rolling out training and education. This was a valuable lesson for hospital. Furthermore, mental healthcare and support services are essential during this period in the fight against COVID-19.
